# P-47. Clinical Impact of Viridans Group Streptococci Bacteremia in Pediatric Oncology and Stem Cell Transplant Patients

**DOI:** 10.1093/ofid/ofaf695.276

**Published:** 2026-01-11

**Authors:** M U A Y A D ALALI

**Affiliations:** Indiana university, Carmel, Indiana

## Abstract

**Background:**

Viridans Group Streptococci (VGS) are associated with significant morbidity and mortality during febrile neutropenia (FN) in pediatric oncology patients; however, pediatric studies from the United States remain limited. This study aimed to describe the clinical characteristics and outcomes of VGS bloodstream infections (VGS-BSI)

**Methods:**

VGS are associated with significant morbidity and mortality during FN in pediatric oncology patients; however, pediatric studies from the United States remain limited. This study aimed to describe the clinical characteristics, outcomes, and resistance patterns of pediatric patients with VGS bloodstream infections (VGS-BSI) undergoing treatment for malignancy or stem cell transplantation (SCT).

**Results:**

Among 2,648 febrile neutropenia episodes in 1,237 patients, 327 bacteremic episodes occurred in 239 patients. VGS was the most frequently isolated pathogen (78/359, 21%), with acute myeloid leukemia (AML) accounting for 33% of VGS-BSI cases, despite acute lymphoblastic leukemia (ALL) being the most common underlying diagnosis overall. Among VGS isolates, 19% were resistant to penicillin and 7% to ceftriaxone/cefepime, though all remained susceptible to vancomycin. Viridans Group Streptococcal Shock Syndrome (VGS-SSS)—defined as hypotension with or without acute respiratory distress syndrome in the absence of another pathogen—occurred in 18 episodes (23%), requiring ICU admission in 14% and resulting in 2 deaths. Although 93% of VGS-BSI cases involved only one day of bacteremia, the mean fever duration was 4 days. Prolonged fever ( >5 days) without an alternative diagnosis occurred in 27%, and 11% had fever lasting >10 days, with 93% resolving after absolute neutrophil count (ANC) recovery. VGS-BSI rates increased steadily from 15% in 2017 to 29% in 2023

**Conclusion:**

VGS bacteremia remains a serious complication in pediatric oncology and SCT patients, particularly those with AML. Emerging resistance to penicillin and cephalosporins is concerning. In clinically stable patients, prolonged fever often resolves with neutrophil recovery and may not require extensive workup. Further research is needed to develop risk stratification tools and guide empirical therapy for those at highest risk of VGS-SSS.

**Disclosures:**

All Authors: No reported disclosures

